# Atorvastatin-Diltiazem Combination Induced Rhabdomyolysis Leading to Diagnosis of Hypothyroidism

**DOI:** 10.1155/2017/8383251

**Published:** 2017-04-11

**Authors:** N. D. B. Ehelepola, S. M. B. Y. Sathkumara, H. M. P. A. G. S. Bandara, K. L. R. Kalupahana

**Affiliations:** The Teaching (General) Hospital-Kandy, Kandy, Sri Lanka

## Abstract

Statins and hypothyroidism, independently, can rarely cause rhabdomyolysis. The combination of them especially with concurrent intake of drugs such as diltiazem increases the risk of rhabdomyolysis. Hashimoto's encephalopathy is a rare condition associated with Hashimoto's thyroiditis and some patients with that can present with a stroke like picture. An elderly male who has been on atorvastatin for three years and on diltiazem for a week presented with sudden onset inability to walk and confusion. On examination muscle tenderness was noticed and creatine kinase levels indicated rhabdomyolysis which we attributed to atorvastatin. Patient developed a seizure and myoclonus of masseters. Considering this, his confusion and his neutrophilia and high C-reactive protein levels, empirical antibiotics with dexamethasone were started and the patient responded to that. His cerebrospinal fluid and blood culture reports that arrived later did not show sepsis. After going home also his CK (creatine kinase) levels remained high; TSH (thyroid-stimulating hormone) level test was done and hypothyroidism was diagnosed. His antithyroid peroxidase antibody levels were also very high. We retrospectively think he had Hashimoto's encephalopathy as well. His lipid profile and TSH and CK values returned to normal in that order after a few months of levothyroxine therapy.

## 1. Introduction

Hashimoto's thyroiditis, the commonest form of hypothyroidism, affects 2–4% of the population [1] and up to 60 percent of people with thyroid diseases are unaware of their disease even in some developed countries [2]. Undiagnosed hypothyroidism per se can cause rhabdomyolysis [3]. According to the World Health Organization in 2008 the global prevalence of raised total cholesterol among adults (≥5.0 mmol/l) was 39% [4] and statins are the mainstay drugs used to control hypercholesterolemia. Undiagnosed hypothyroidism can present as hypercholesterolemia. Cytochrome P450 (CYP) 3A4 is the most abundant enzyme of CYPs in the liver and intestines that metabolize approximately 50% of currently available drugs including statins [5]. CYP 3A4 inhibitors including calcium channel blockers like diltiazem are also widely prescribed and they increase plasma levels of statins [6, 7]. Therefore simultaneous intake of calcium channel blockers with statins increases the risk of developing rhabdomyolysis [6]. This risk is further increased by concurrent undiagnosed hypothyroidism [6]. Many doctors known to us prescribe the combination of statins and diltiazem commonly. During the 1996–2012 period, atorvastatin became the best-selling drug in the human history [8]. Despite the aforesaid background, lack of case reports of atorvastatin-diltiazem and undiagnosed hypothyroidism combination induced rhabdomyolysis indicates this important problem is underdetected and underreported. However, our literature survey found one case report of rhabdomyolysis due to atorvastatin-diltiazem combination [9].

Hashimoto's encephalopathy (HE), also known in other names, is a rare syndrome associated with Hashimoto thyroiditis with high antithyroid peroxidase (TPO) antibody levels [10, 11]. It is often characterized by acute or subacute onset of confusion with altered level of consciousness, seizures, and myoclonus [10, 11]. It can present as a stroke like pattern or slowly progressive cognitive impairment, but these patterns can overlap [10]. HE responds well to steroids [10, 11].

## 2. Case Report

A 72-year-old average-built male presented with the first attack of sudden onset weakness of both legs and inability to walk, impaired memory and confusion, and abnormal behavior for three days. He has been on treatment for hypertension and dyslipidemia during the previous three years. At the time of admission he was on losartan, aspirin, hydrochlorothiazide, diltiazem, and atorvastatin. There was no history of fever, any trauma or strenuous exertion in the recent past, diabetes, thyroid diseases, backache, seizures, slurring of speech, numbness, or loss of sensations of limbs. He was used to consuming alcohol approximately once a month. On examination his pulse rate was 70/minute; blood pressure was 130/80 mm Hg; cardiovascular, respiratory, abdominal, and nervous system examinations were unremarkable except muscle power was 4/5 and all tendon reflexes were diminished in all four limbs. Tenderness of muscles was noticed on examination which aggravated towards the third day at hospital and then gradually improved and subsided by the eighth day. However, he complained of muscle pain only from day 2 at hospital.  Table 1 shows his laboratory investigation results.

His noncontrast computed tomography of brain was normal. Initial laboratory investigation results showed hyponatremia and hypokalemia; hence hydrochlorothiazide was omitted. We initially assumed hyponatremia as the cause of his confusion and corrected that with normal saline infusions.

His initial CK level was >18 times higher than the normal upper limit. Atorvastatin induced rhabdomyolysis was diagnosed and that drug was stopped. Intravenous normal saline 2-3 l per day was infused and adequate urine output was maintained. His hypokalemia was corrected with intravenous KCl infusions. On the third day he developed one attack of convulsions lasting for <5 minutes. On day 4 we started empirical intravenous ceftriaxone and dexamethasone considering his persisting confusion, neutrophilia and high CRP levels, and previous day convulsions. However, there were no fever and signs like neck stiffness. Those two drugs were continued for seven days while waiting for reports of CSF obtained by lumbar puncture, blood, and urine culture reports. Those reports did not show evidence of any infection. However CSF protein level was 70 mg/dL. Patient had an episode of myoclonus of masseter muscles and generalized increase of muscle tone on the same day (day 4) that subsided on the following day; nerve conduction velocity studies showed predominantly sensory axonal polyneuropathy and marginally low motor conduction velocity. Electro encephalogram (EEG) showed low amplitude cortical activity in slower range. Patient's muscle tenderness gradually reduced; he was able to walk and became rational and well oriented and on day 8 he was sent home and asked to come to our clinic in a week for a review.

After a week the patient was rational and well oriented and could walk. The CK level was 213 U/l, but it again increased and has not decreased below 386 U/l when the test was repeated later in spite of not being on atorvastatin. He was reassessed; considering CK levels, his hyponatremia and hypokalemia, and slow relaxing ankle jerk, hypothyroidism was suspected. When asked him to think carefully and answer; he admitted that he felt lethargic and there were episodes of impaired memory on and off for about 10 months before his acute illness. He had mentioned his memory impairment to his general practitioner (GP) during his last visit. But there was no voice change, constipation, weight gain, changes of skin or libido, hair loss in the recent past, cold intolerance, or joint pains. And he insisted his inability to walk and confusion were of sudden onset and his wife vouched for him. He had very high TSH levels (86.1 mIU/l, normal range 0.3–4.2 mIU/l). Hypothyroidism was diagnosed. Levothyroxine 25 micrograms (*μ*g) per day was started and gradually increased up to 75 *μ*g per day. His (TPO) antibody level was >1000 IU/mL (normal < 35 IU/mL) and that test was repeated and confirmed. Further literature survey by first author enlightened us of risk of atorvastatin-diltiazem combination and the likelihood of him having Hashimoto's encephalopathy also while he was hospitalized.

His TSH levels reduced progressively but not the CK levels. After levothyroxine intake was increased above 100 *μ*g per day, CK levels also started to decrease to normal level gradually.

His lipid profile done two months after starting levothyroxine was normal. After 10 months his TSH, CK, and lipid profiles were normal; he was healthy except for a discoid eczematous rash developed in limbs that responded to topical steroids.

## 3. Discussion

This patient presented to us with history more suggestive of a neurological problem. He did not complain of myalgia on or before admission. Detection of muscle tenderness on physical examination and then a very high CK levels directed us to diagnose statin induced rhabdomyolysis. He had confusion, hyponatremia, and myalgia but denied history of any thyroid problems; other common symptoms of hypothyroidism were not there and there was no goiter. We relied on that and did not order TSH test earlier. Persistence of his high CK levels long after stopping atorvastatin compelled us to rethink which led us towards the diagnosis of underlying hypothyroidism. In younger patients with dyslipidemia we and most doctors known to us try to exclude hypothyroidism but this case shows there may be a place for doing that even in the case of older patients if there is some suspicion. However, in countries with limited resources like ours we have to limit the laboratory investigations. It is well known that hypothyroidism has diverse and unusual and uncommon presentations. This is a very unusual presentation.

This patient has been on treatment from a GP for hypertension and dyslipidemia. He has been on atorvastatin for three years. However he did not visit his GP regularly. For example, during the year preceding admission to the hospital, for 10 consecutive months he purchased the same drugs from a pharmacy showing the same old prescription. It is a common practice in Sri Lanka to avoid doctors' fees in the private sector or to avoid long waiting and congestion of state hospitals like ours that provide free service including drugs. That may have contributed to nondetection of his hypothyroidism earlier. Then he changed his GP who replaced metoprolol of his drug list with diltiazem. We presume that patient's complaining of memory impairment to his new GP led to replacement of metoprolol. One week after that, the combination of undiagnosed hypothyroidism, atorvastatin, and diltiazem would have caused his rhabdomyolysis.

There is one case report from China about rhabdomyolysis induced by simvastatin-diltiazem interaction in unrecognized hypothyroidism [6]. In contrast to our patient he has been on simvastatin for about a year; one month before presentation diltiazem was started and he presented with myalgia for 25 days [6]. Considering that both statins and calcium channel blockers are prescribed very commonly, undiagnosed hypothyroidism is also common and rhabdomyolysis is life-threatening; this case would be a valuable reminder to clinicians.

This patient's very high anti-TPO antibody levels indicate that he has Hashimoto's disease. His sudden onset of presenting symptoms (that mimic a cerebrovascular event with confusion) of seizure and myoclonus at the hospital and normal CSF report except high protein level all point towards Hashimoto's encephalopathy [10, 11]. We suspected those symptoms were of possible meningoencephalitis and gave him intravenous dexamethasone adjuvant to ceftriaxone to prevent further neurological sequelae and that may have resulted in him recovering from Hashimoto's encephalopathy [10]. As he well responded to that treatment and even CSF and blood culture reports did not show evidence of any sepsis, we continued that treatment. Looking retrospectively we think it is likely that had a concurrent attack of HE (not meningoencephalitis) that well responded to dexamethasone. Hyponatremia and hypokalemia which we initially suspected as reasons for his symptoms can be associated with HE [12]. Nonetheless HE usually need longer course of steroids to recover and our patient did not get recurrences during the following 14 months as about half of HE patients do [11].

This may be the first reported case of undiagnosed hypothyroid patient presenting with concurrent probable Hashimoto's encephalopathy and atorvastatin-diltiazem combination induced rhabdomyolysis. This case reiterates the importance of knowledge of drug interactions in day to day practice and the need to be cautious of potential myopathy when prescribing statins to hypothyroid patients, especially in combination with cytochrome P450 3A4 inhibitors. This case exemplifies the risks of buying drugs from pharmacies repeatedly using old prescriptions without being seen by a doctor. This illustrates importance of looking for possible drug interactions and hypothyroidism in the cases of statin induced myositis/rhabdomyolysis as well. For grey cases of meningitis with seizures we start antibiotics with dexamethasone pending CSF reports. If the patient improves even if the report comes negative, we presume our clinical diagnosis as correct. This case demonstrates we have to consider rare possibilities like HE also in such instances.

## Figures and Tables

**Table 1:** The summery of the laboratory workup results of this patient.

Investigation	Reference range	Value/the day at hospital on which the test was done is within brackets

Creatine kinase (CK) in U/l	*38–174*	*3169.5 (1)→2382.4 (3)→1233.0 (5)*
White cell count (×10^9^/l)	*4.0–10.0*	*10.7 (1)→11.84 (4)*
Neutrophils %	*50–70*	*76.7% (1)→88.6 (4)*
Lymphocytes%	*20–40*	*17.3% (1)→8.4 (4)*
Hemoglobin (g/dL)	*11–16*	*14.1 (1)→11.2 (4)*
Hematocrit (%)	*37–54*	*42% (1)→32 (4)*
Platelets count (×10^9^/l)	*150–450*	*145*
Reticulocyte count	*0.5–1.5%*	*2.6% (4)*
Serum alanine transaminase (ALT) in U/l	*7.0–28*	*77 (1)*
Serum aspartate transaminase (AST) in U/l	*13.0–31.0*	*229 (1)→210 (3)*
Serum alkaline phosphatase (ALP) in U/l		*207.2→210*
Serum C-reactive protein (CRP) in mg/l		*5.2 (1)→144.2 (4)→28 (9)*
Blood urea (mmol/l)	*2.1–7.1*	*6.0 (1)→6.8 (2)→8.4 (3)→7.0 (6)*
Serum creatinine (mg/dL)	*0.9–1.30*	*1.25 (1)→1.0 (2)→1.25 (3)→1.28 (4)→1.20 (6)→0.89 (8)*
Serum sodium (mmol/l)	*133–148*	*110 (1)→126 (1)→130 (2)→116 (3)→116 (4)→130 (5)→127 (6)→130 (7)→137 (8)*
Serum potassium (mmol/l)	*3.5–5.3*	*2.6 (1)→4.0 (1)→5.0 (2)→2.6 (3)→2.3 (4)→2.1 (5)→3.9 (6)→3.1 (7)→3.5 (8)*
Serum calcium (mmol/l)	*2.10–2.55*	*2.07 (1)→2.04 (2)→2.35 (5)*
Urine full report		*Pus cells 5 per HPF, Red Cells 8–10 per HPF and Albumin + (2)*
ESR (mm/hour)	*<20*	*7 (2)*
Fasting blood sugar (mg/dL)	*70–126*	*108 (2)*
Blood culture		*No growth (4)*
Urine culture		*No growth (4)*

## Abbreviations

CRP: C-reactive protein
CSF: Cerebrospinal fluid
CK: Creatine kinase
TPO: Thyroid peroxidase
CYP: Cytochrome P450
HE: Hashimoto's encephalopathy
EEG: Electro encephalogram
GP: General practitioner
TSH: Thyroid-stimulating hormone.
